# Procedures performed by general practitioners and general internal medicine physicians - a comparison based on routine data from Northern Germany

**DOI:** 10.1186/s12875-018-0878-3

**Published:** 2018-12-03

**Authors:** C. Strumann, K. Flägel, T. Emcke, J. Steinhäuser

**Affiliations:** 1 Institute of Family Medicine, University Hospital Schleswig-Holstein, Campus Luebeck, Ratzeburger Allee 160, 23538 Luebeck, Germany; 2 Association of Statutory Health Insurance Physicians of the Federal State of Schleswig-Holstein, Bismarckallee 1-6, 23795 Bad Segeberg, Germany

**Keywords:** Procedural skills, Primary care, General internal medicine, General practitioner

## Abstract

**Background:**

In response to a rising shortage of general practitioners (GPs), physicians in general internal medicine (GIM) have become part of the German primary care physician workforce. Previous studies have shown substantial differences in practice patterns between both specialties. The aim of this study was to analyse and compare the application of procedures by German GPs and GIM physicians based on routine data.

**Methods:**

The Association of Statutory Health Insurance Physicians in the federal state Schleswig-Holstein (Northern Germany) provided invoicing data of the first quarters of 2013 and 2015. Differences between GPs and GIM physicians in the implementation rate of 46 selected primary care procedures were examined by means of the Pearson *χ*^2^-test. The selection of procedures was based on international and own preliminary studies on primary care procedures.

**Results:**

In the first quarter of 2013/2015 respectively, 1228/1227 GPs and 447/484 GIM physicians provided services in Schleswig-Holstein. Significant differences were found for 20 of the 46 procedures. GPs had higher application rates of procedures concerning health screening (e.g. adolescent health examination, well-child visits) and minor surgery. GIM physicians more often applied technology-oriented procedures, such as ultrasound scans, electrocardiograms (ECG), and 24-h ambulatory blood pressure measurements. The treatment patterns of both specialities did not vary much during the study period. Cardiac stress testing was the only significantly increased GP procedure in that time.

**Conclusions:**

Our results suggest substantial differences in the application of procedures between GPs and GIM physicians with potential consequences for the overall primary healthcare provision. The findings could foster a discussion about training needs for procedures in primary care to ensure its comprehensiveness. The results reflect scope for changes in vocational training in the future for an effective and efficient re-allocation of primary healthcare.

## Background

In most industrialised countries, the demand for healthcare is increasing due to an ageing population coinciding with a declining number of primary care physicians [1–3]. Primary care physicians are general practitioners (GPs), physicians in general internal medicine (GIM) or paediatricians who provide “care for the undifferentiated patient at the point of first contact” [4]. The range of services that primary care patients require is extensive [5]. However, according to previous studies, procedures differ considerably between GPs and GIM physicians, e.g. regarding the use of diagnostics [6], medical charges [7], prescribing [8], communication [9], provision of care for patients with common conditions [10], range of specific health needs covered [11] and patient outcomes [12].

In Germany, most primary care physicians are traditionally self-employed. However, they need an accreditation for service provision for patients within the statutory health insurance scheme that covers about 90% of the population. In 2016, around 79% of the German population visited a primary care physician at least once [13]. The distribution of physicians is regulated and allocated by the Association of Statutory Health Insurance Physicians (ASHIP), which is responsible for the accreditation process to maintain a sufficient and high-quality supply of physicians [14].

Whereas the international definition of primary care physicians includes paediatricians, the definition by the German ASHIP does not, so that the primary care physician workforce in Germany only consists of GPs and GIM physicians. By law, GPs are preferred in the accreditation process [15]. However, the declining number of GPs in the last decade has resulted in local shortages, especially in rural areas [16, 17]. In response to the rising shortage of GPs, a rise in the quota of GIM physicians providing primary care can be observed [18].

A previous study using self-assessment of GPs and GIM physicians [19] showed differences in the application of medical procedures and suggested implications for the quality and safety of primary care provision in Germany. In general, the use of survey data based on self-assessment can be problematic because of selection or response biases [20]. Influences on self-assessment such as gender, age, emotional status and recall bias have been described [21–24]. Moreover, studies based on survey data are prone to selection bias [25]. In contrast, routine data present a reliable source of information that avoid selection or recall bias [26, 27]. Findings of studies based on survey data can be crosschecked by analysing routine data.

The aim of this study was to analyse and compare, based on routine data, the application of procedures by German GPs and GIM physicians. The results may subsequently allow to deduce measures to promote an effective and efficient re-allocation of primary healthcare resources.

## Methods

This study is based on the analysis of routine data from the ASHIP of the federal state Schleswig-Holstein located in Northern Germany. The concentration on a specific federal state of Germany allows to reduce practice variations based on regional differences and state-specific regulations [28].

### Data selection

Based on a previously consented questionnaire comprising relevant procedures in German primary care [19, 25] the research team checked the doctor’s fee scale 2015 [29] for codes addressing procedures or at least comprising procedures of the questionnaire. Both the routine data provided by the ASHIP and the data collected by the questionnaire refer to the first three months of the years 2013 and 2015 respectively. Data included the number of all billed codes of the doctor’s fee scale by all GPs and GIM physicians in the federal state of Schleswig-Holstein. The analysis concentrated on codes reflecting services with high relevance in primary care [30–33]. Procedures were defined as discrete, diagnostic or therapeutic activities requiring knowledge and manual skills, performed on patients following the definition of Sylvester et al. [31]. Out of 90 possible physicians’ procedures listed in the initial questionnaire [19, 25], 46 were identified that could be employed by both GPs and GIM physicians. Table 1 shows the codes for services and whether the respective procedure is part of the training curriculum of the respective specialty.

**Table 1:** Assignment of the procedures to the bill codes

Code	Bill code description [29]	Procedure [25]	Part of training [45]	
			GP	GIM
Well-child visit				
01712	Well-child visit of the new-born during the 3rd to 10th day of life	Well-child visit	yes	no
01713	Well-child visit during the 4th and 5th week of life “(U3)”			
01714	Well-child visit during the 3rd and 4th month of life “(U4)”			
01715	Well-child visit during the 6th and 8th month of life “(U5)”			
01716	Well-child visit during the 10th and 12th month of life “(U6)”			
01717	Well-child visit during the 21st to 24th month of life “(U7)”			
01718	Well-child visit during the 46th to 48th month of life “(U8)”			
01719	Well-child visit during the 60th to 64th month of life “(U9)”			
01723	Well-child visit during the 36th to 43rd month of life “(U7a)”			
01720	Adolescent health examination “(J1)”	Adolescent health examination	yes	no
Minor surgery				
02301	Minor surgery II: primary wound closure with sutures	Surgical suture	yes	no
		Drainage of acute paronychia	yes	no
		Electrocautery of skin lesion	yes	no
		I&D of an abscess	yes	no
		I&D of a perianal abscess	yes	no
		Glueing of a wound	yes	no
02302	Minor surgery III: excisions, treatment of ingrown toenails, phlebotomy	Removal of foreign object	yes	no
		Excision of lipoma	yes	no
		Partial removal of toenail	yes	no
02310	Secondary healing wound care and/or decubital ulcer care	Wound debridement	yes	no
02311	Diabetic foot care			
02312	Treatment of single or multiple chronic venous ulcers			
02313	Compression therapy for chronic venous insufficiency, post-thrombotic syndrome, superficial and deep vein thrombosis and/or lymphoedema	Compression therapy	no	no
Injection and infusion				
02101	Infusion	Infusion	yes	yes
02321	Suprapubic catheter insertion	Suprapubic catheter insertion	yes	yes
02323	Transurethral catheter insertion	Transurethral catheter insertion	yes	yes
Paracentesis and insertion				
02340	Paracentesis	Ascites paracentesis	yes	yes
		Trepination of subungual haematoma	yes	no
		Paracentesis of knee joint	yes	yes
		Paracentesis of scapula joint	yes	yes
02343	Trephination of pleural cavity and non-surgical pleural drainage	Thoracentesis	yes	yes
		Trepination of tension pneumothorax	yes	yes
		Chest tube insertion	yes	yes
Treatment of musculoskeletal disorders				
02360	Treatment under local anaesthesia	Neural therapy	yes	no
31,910	Reduction of carpal or tarsal dislocation (distal)	Reduction of dislocated finger	no^a^	no
31,912	Reduction of dislocated cubital or knee joint (distal)	Reduction of displaced fracture of the radial head	no^a^	no
31,914	Reduction of dislocated cubital or knee joint (proximal)	Reduction of dislocated shoulder joint	no^a^	no
Instrument-based procedures				
02500	Single inhalation therapy	Preparing a nebulizer for antiobstructive therapy	no	no
03321	Cardiac stress test	Cardiac stress test	yes	yes
03324	24-h ambulatory blood pressure monitoring	24-h ambulatory blood pressure monitoring	yes	yes
03330	Spirometry	Spirometry	yes	yes
03331	Proctoscopy	Proctoscopy	yes	yes
03335	Exploratory audiometry after previously documented, hearing test anomalies	Diagnostic audiometer test	no	no
33012	Thyroid sonography B-scan	Thyroid sonography thyroid	yes	yes
33042	Abdominal sonography B-scan	Abdominal sonography	yes	yes
33060	Sonographic examination of extracranial cerebral vessels, the periorbital arteries, subclavian arteries and vertebral arteries by CW-Doppler	Doppler ultrasound of brain-supplying vessels	yes	yes
33061	CW Doppler sonography of limb blood vessels, at least 3 transducer locations per limb	Compression sonography of lower extremities	yes	yes
33076	Limb vein B-scan sonography at least 8 transducer locations			
Laboratory diagnostic procedures				
32031	Microscopic urinalysis for morphological components	Microscopic urinalysis	yes	yes
32040	Faecal occult blood test in 3 samples	Faecal occult blood test	yes	yes
01734	Examination for faecal occult blood according to stage D.-III of the early detection of cancer-guideline, including costs			
32045	microscopic examination of bodily material	Examine a native sample for funghi	yes	yes
Emergency medicine				
01220	Resuscitation	Mask ventilation	yes	yes
01221	Supplement to Resuscitation (Coniotomy and / or Endotracheal Intubation)	Endotracheal intubation	yes	yes
01222	Supplement to Resuscitation (defibrillation)	Defibrillation	yes	yes
Gynaecology				
01830	Insertion of intrauterine device	insertion of intrauterine device	no	no
01730	Cancer screening for women	Gynaecological examination	no	no
01825	Cervical smear test	Cervical smear test	no	no

^a^These procedures are part of the mandatory 6-month surgical training during GP vocational training

### Statistical analysis

Differences in the application of a specific procedure by GPs and GIM physicians as well as differences between the two study periods were analysed by means of the Pearson *χ*^2^-test. All tests of significance were two-tailed and were corrected using the Bonferroni method to counteract the problem of multiple comparisons [34]. A *p*-value < 0.05 was considered as statistically significant. Statistical analyses were performed with MATLAB software, version 9.4 (R2018a) (The MathWorks, Natick, MA, USA).

## Results

In the first quarter of 2013, 1228 GPs and 447 GIM physicians provided services in Schleswig-Holstein. In 2015, the number of GPs remained unchanged (1227), while the number of GIM physicians had increased to 484. There are no substantial differences between the number of distinct fee scale codes submitted by GPs and GIM physicians. For both specialities the overall number of services invoiced and the physicians’ average of services invoiced have increased over the time frame by 37.7% and 27.2%, respectively. The percentages of the total number of codes reflecting the selected procedures are relatively small and have declined slightly over the study period (2015: 2% (GP) and 2.7% (GIM)). Table 2 shows the number of physicians, the number of distinct service codes, the total number of codes invoiced and other statistics for both years and specialties.

**Table 2:** Descriptive Statistics

year	2013		2015		change (in %)	
specialty	GP	GIM	GP	GIM	GP	GIM
physicians	1228	447	1227	484	−0.1	8.3
number of fee scale codes invoiced	697	613	683	675	−2.0	10.1
total number of codes invoiced by all physicians	6,752,667	2,499,625	8,856,848	3,442,080	31.2	37.7
total number of codes invoiced per physician	5498.9	5592.0	7218.3	7111.7	31.3	27.2
total number of codes invoiced by all physicians reflecting selected procedures	179,095	85,413	180,779	93,286	0.9	9.2
percentage of the total number of codes reflecting selected procedures (in %)	2.7	3.4	2.0	2.7	−23.0	−20.7

There are several significant differences between GIM physicians and GPs with regard to the application of specific procedures. Of note are the higher GP figures for health screening services, especially adolescent health examination and well-child visits. The number of minor surgery procedures performed by GPs is also significantly higher. These include primary and secondary wound healing, excisions, treatment of ingrown toenails and phlebotomy. Procedures performed to a higher extent by GMI physicians are in general based on more technical approaches, i.e. services using ultrasound diagnostics or an electrocardiogram (ECG). Another service that is significantly more frequently performed by GIM physicians than GPs is the long-term blood pressure measurement. In general, the treatment patterns of both specialties did not vary much over the time frame. Cardiac stress testing was the only procedure by GPs that saw an increase in the study period. The proportions of GPs and GIM physicians performing a specific procedure in the study periods of 2013 and 2015 are shown in Tables 3 and 4 with the respective *p*-values for group differences. For ease of illustration, procedures which were applied by less than 1% of physicians are not shown.

**Table 3:** Application rates of general practitioners (GPs) and general internal medicine (GIM) physicians for procedures performed to a greater extent by GPs (in %)

Code	Description	2013			2015		
		GP (*n* = 1228)	GIM (*n* = 447)	*p*-value*	GP (*n* = 1227)	GIM (*n* = 484)	*p*-value*
**02310**	Secondary healing wound care and/or decubital ulcer care	60.4	43.6	< 0.001	64.6	44.0	< 0.001
**02301**	Minor surgery II: primary wound closure with sutures	39.0	13.4	< 0.001	37.8	16.7	< 0.001
**01734**	Examination for faecal occult blood according to stage D.-III of the early detection of cancer-guideline, including costs	27.2	22.1	n.s.	27.9	24.8	n.s.
**01720**	Adolescent health examination “(J1)”	27.1	13.9	< 0.001	25.5	9.5	< 0.001
**02312**	Treatment of single or multiple chronic venous ulcers	23.2	17.4	n.s.	23.5	17.8	n.s.
**01718**	Well-child visit during the 46th to 48th month of life “(U8)”	13.8	2.5	< 0.001	12.2	1.2	< 0.001
**01719**	Well-child visit during the 60th to 64th month of life “(U9)”	13.6	2.0	< 0.001	13.9	1.4	< 0.001
**02302**	Minor surgery III: excisions, treatment of ingrown toenails, phlebotomy	13.0	2.0	< 0.001	12.1	2.1	< 0.001
**01723**	Well-child visit during the 43rd to 36th month of life “(U7a)”	10.9	1.6	< 0.001	11.8	1.7	< 0.001
**01717**	Well-child visit during the 21st to 24th month of life “(U7)”	10.6	0.9	< 0.001	10.2	1.0	< 0.001
**01716**	Well-child visit during the 10th and 12th month of life “(U6)”	10.3	1.8	< 0.001	8.6	1.2	< 0.001
**01715**	Well-child visit during the 6th and 8th month of life “(U5)”	8.3	1.3	< 0.001	8.2	1.2	< 0.001
**01714**	Well-child visit during the 3rd and 4th month of life “(U4)”	8.1	1.1	< 0.001	7.1	1.2	< 0.001
**01713**	Well-child visit during the 4th and 5th week of life “(U3)”	6.4	0.9	< 0.001	6.4	0.6	< 0.001
**02500**	Single inhalation therapy	5.3	2.5	n.s.	4.9	2.3	n.s.
**03335**	Exploratory audiometry after previously documented, hearing test anomalies	3.7	1.6	n.s.	3.1	0.8	n.s.
**01730**	Cancer screening for women	3.2	0.0	0.010	1.8	0.0	n.s.
**01712**	Well-child visit of the new-born during the 3rd to 10th day of life	2.5	0.0	n.s.	2.4	0.2	n.s.

*Bonferroni correction

only procedures with percentages larger than 1.0% for at least one specialty are shown

bold percentages indicate a significant difference between 2013 and 2015 (at the 5% level)

n.s. not significant (at the 5% level)

**Table 4:** Application rates of general practitioners (GPs) and general internal medicine (GIM) physicians for procedures performed to a greater extent by GIM physicians (in %)

Code	Description	2013			2015		
		GP (*n* = 1228)	GIM (*n* = 447)	*p*-value*	GP (*n* = 1227)	GIM (*n* = 484)	*p*-value*
**03330**	Spirometry	80.5	82.3	n.s.	80.1	83.7	n.s.
**03324**	24-h ambulatory blood pressure monitoring	72.2	82.8	< 0.001	74.2	82.9	0.011
**32040**	Faecal occult blood test in 3 samples	68.5	70.0	n.s.	66.0	66.9	n.s.
**33042**	Abdominal sonography B-scan	52.9	91.1	< 0.001	51.6	89.3	< 0.001
**03321**	Cardiac stress test	**45.5**	79.6	< 0.001	**52.6**	82.4	< 0.001
**32031**	Microscopic urinalysis for morphological components	37.8	41.6	n.s.	34.5	34.9	n.s.
**33012**	Thyroid sonography B-scan	21.4	73.6	< 0.001	21.5	75.4	< 0.001
**02313**	Compression therapy for chronic venous insufficiency, post-thrombotic syndrome, superficial and deep vein thrombosis and/or lymphoedema	15.8	16.1	n.s.	20.7	19.8	n.s.
**03331**	Proctoscopy	6.4	7.4	n.s.	5.2	7.2	n.s.
**02311**	Diabetic foot care	6.2	10.1	n.s.	5.5	9.9	n.s.
**33061**	CW Doppler sonography of limb blood vessels, at least 3 transducer locations per limb	1.6	8.5	< 0.001	1.5	7.2	< 0.001
**33060**	Sonographic examination of extracranial cerebral vessels, the periorbital arteries, subclavian arteries and vertebral arteries by CW-Doppler	1.1	3.6	0.036	1.1	3.3	n.s.
**01220**	Resuscitation	0.7	1.1	n.s.	1.1	0.0	n.s.
**33076**	Limb vein B-scan sonography at least 8 transducer locations	0.3	8.7	< 0.001	0.6	9.3	< 0.001

*Bonferroni correction

only procedures with percentages larger than 1.0% for at least one specialty are shown

bold percentages indicate a significant difference between 2013 and 2015 (at the 5% level)

n.s. not significant (at the 5% level)

## Discussion

The comprehensiveness of general practice in the provision of primary healthcare [35] and its coordinating role in referring patients across the individual healthcare sectors [36, 37] determine the strength of primary care, since both factors have positive effects on health outcomes, equality and overall efficiency in healthcare systems [38–43]. Therefore, GPs traditionally received training focusing on treating the whole person through all stages of life [44]. The curriculum of GPs in Schleswig-Holstein includes working in primary care, i.e. private practices for at least 24 months. In contrast, the training of internal medicine physicians happens entirely in the hospital setting [45].

We analysed differences in patterns of procedures performed by German GPs and GMI physicians based on routine data collected by ASHIP for the federal state of Schleswig-Holstein over two distinct time periods in 2013 and 2015. In total, 1227 GPs and 484 GIM physicians were looked at in 2015. This cohort represented about 3.5% of all GPs and GIM physicians practising in Germany in 2015 [18]. The ratio of codes billed per specialty (GPs: 72% (73%) and GIM physicians: 28% (27%) in 2015 (2013)) are nearly identical for both specialties and years to the respective nation-wide proportions of cases treated and codes billed in primary care [46, 47]. Furthermore, the differences over the time frame underline the rising significance of GIM physicians in the provision of primary care in Germany.

The results show substantial differences between GPs and GIM physicians in the application rates of most of the identified procedures. In general, procedures with higher application rates by GPs tend to be more advisory and concern the prevention of health problems. In contrast, procedures with higher application rates by GMI physicians are to a greater extent technically orientated. These results are consistent with findings of previous studies [7, 12, 19].

In 2015, for example, more than 25% of GPs performed health visits for adolescents. In contrast, less than 10% of GIM physicians performed this procedure. Well-child visits show similar differences. These figures reflect a wider range in the age of patients treated by GPs compared to GIM physicians. Health services for children are also provided by paediatricians [48]. In German rural areas, however, a shortage of paediatricians leads to children’s healthcare services being delivered by GPs or GIM physicians [49]. Moreover, rural areas are particularly affected by the declining number of GPs. Therefore, GIM physicians stand a higher chance of accreditation in rural areas to counteract the shortage of GPs. Our results suggest that GMI physicians practising in rural areas need to get involved in children’s and adolescents’ healthcare services in order to safeguard a high quality of service provision.

Some of the procedures dealing with health screening of adults have also significantly higher application shares for GPs. This is in line with findings of previous studies, that GPs place more emphasis on preventive services [7, 12]. Recent studies highlight the positive effects of preventive care on the reduction of hospital admissions and emergency department visits [50–52]. In Germany, the increasing number of non-urgent emergency department visits has resulted in overstretched emergency facilities with negative effects on quality and effectiveness of the emergency care provision [53–55]. Especially in rural areas with relatively high proportions of elderly people [56] and limited public transport to gain access to primary healthcare provision [57] emergency departments tend to compensate for the lack of primary care physicians [58, 59]. This study found that a significantly smaller percentage of GIM physicians provided preventive services. Therefore, an increase in the overall number of GIM physicians is likely to result in a decrease in the provision of preventive services. This points to a need for the inclusion of preventive medicine in the curriculum for the future training of GIM physicians and postgraduate training for GIM physicians, especially for those practising in rural areas.

Most of the procedures involving minor surgery show significantly higher application rates for GPs. Minor surgery is largely part of the vocational training scheme of GPs as opposed to the training of GIM physicians [45]. Advantages of providing minor surgery in a primary care setting include improved access to surgical care for patients, reduced waiting times and improved patient satisfaction [60]. Although evidence about the quality and cost effectiveness is mixed [61], there are international studies that suggest lower referral rates to secondary care if minor surgery procedures had been performed by GPs [62]. Furthermore, only small differences have been observed between the quality of minor surgery procedures carried out in primary and secondary care setting. In any case, patient satisfaction for minor surgery procedures performed in primary care has been higher [63]. The data show a slightly but insignificant increase over time of GIM physicians offering minor surgery procedures, resulting in a narrowing of the gap between both specialties. However, in order to boost this development, a reassessment of GIM physicians’ vocational training programmes should focus on minor surgical procedures.

Similar to previous findings [7, 12, 64] a more technical orientation of GIM physicians was observed in this study. Moreover, GIM physicians had significantly higher shares for procedures that, in Germany, are close to the specialty of internal medicine, e.g. ultrasound diagnostics, cardiac stress testing and 24-h blood pressure monitoring. This is not surprising due to the exclusive emphasis on internal medicine in the GIM physicians’ training. A previous study showed that German GPs practising in rural areas perform a larger number of distinct procedures compared with GPs in urban areas [25], indicating that they may offset a lack of specialists. This is in line with the findings of Starfield et al. [11] that patients in the US who have a GP as their primary care physician see fewer specialists. The ability to perform a wider range of specialist procedures should be trained for both GPs and GIM physicians, especially for those who practise in rural areas.

In summary, our results suggest substantial differences in the application of procedures between GPs and GIM physicians with potential consequences for primary healthcare provision in general. Most of the differences correlate with differences in the training programmes. As only about 30% of the consultations in primary care practice relate to internal medicine [65], the findings could foster a discussion about training needs for procedures in primary care to ensure its comprehensiveness. The results reflect scope for changes in vocational training in the future. On the other hand, GIM physicians have the opportunity to train their procedural skills, e.g. by attending the educational seminars accompanying the post-graduate training for GPs. These seminars have been defined by the German College of General Practice and Family Physicians (DEGAM) as a core element to improve trainees’ specific knowledge and competencies [66]. From the beginning, the trainees attend training courses preparing for the specific requirements of independent medical work, especially in rural regions.

Beyond training, economic incentives may also reduce the differences in the application of procedures between GPs and GIM physicians. In general, the use of financial incentives is considered to control the physician’s behaviour [67, 68]. Improving the billing options and financial rewards for specific procedures (e.g. preventive care or minor surgeries) might encourage primary care physicians to perform these procedures more often. The explicit effect of changes in the reimbursement on the application of procedures is an interesting issue for future research.

### Strengths and limitations

The study highlights the difference in services provided by GPs and GIM physicians and provides suggestions about emphases for residency trainings and future efforts for an effective and efficient re-allocation of primary healthcare.

The study has strengths as well as limitations. A strength of this study is that it relies on routine data collected for all GPs and GIM physicians in a specific region of Germany. There are no issues related to any selection or response bias, as might be the case when survey data are used [20]. Social desirability bias might play a particular role when surveying physicians about their services. On the one hand, focusing on the federal state of Schleswig-Holstein constrains the representativeness of the findings, on the other hand, this reduces practice variations based on regional differences and state-specific regulations [28]. Furthermore, this study shows only unconditional differences between both specialties. The age, gender, experience and regional characteristics of the physician may also determine the probability of specific services being provided [25]. Moreover, we cannot control for the patient mix. Unfortunately, for Germany there is no evidence available about the differences in the patient mix between GPs and GIM physicians. In Germany, patients can freely choose their doctor. Although an increasing use of physician-rating websites can be observed [69, 70] most patients consult the nearest primary care physician [66]. For example, in the US, only half of the primary care patients know whether their doctor has been trained as a GP or GIM physician [67]. Therefore, we conclude that the specialty of the primary care physician does not have a strong effect on the patient’s physician choice in Germany either. Moreover, the study considered differences between the percentages of physicians performing a specific procedure even when only performed once. This measure will be relatively robust against moderate differences in the patient mix. However, future studies analysing practice style patterns of GIM physicians and GPs should be based on a country-wide dataset and take into account regional and personal characteristics, as well as patient mix information.

Another limitation is that most of the procedures applied by GIM physicians and GPs are not directly represented by the schedule of service codes. This has resulted in a limited number of procedures that were analysed. Moreover, opportunistic practices to increase the reimbursement might lead to billing of services that are not actually performed. Another limitation is given by potential differences between GPs and GIM physicians in their knowledge about how to bill specific procedures. Since GIM physicians do not necessarily need to pass through training in private practices, they may have different awareness or prioritisation in regard to billing. However, in comparison with the use of survey data about procedures applied and the related problems mentioned above, these issues may be negligible.

A further limitation is that the ASHIP is in charge only for the reimbursement of services that are provided to patients within the statutory health insurance system. Services provided to privately insured patients are not covered by the underlying dataset. There are large differences between the service provision for privately and statutorily insured patients [71]. Since the dataset covers 85% of the population of Schleswig-Holstein [72], this is regarded as a minor limitation. However, the effect of the health insurance status on service provision may represent an interesting topic for future research.

## Conclusion

This study shows substantial differences in the application of procedures between GPs and GIM physicians with potential consequences for the overall primary healthcare provision. Most of the differences are explainable with differences in the training programmes between both specialties. These findings could foster a discussion that primary care physicians should uniformly master relevant procedures in primary care and reflect scope for changes in vocational training in the future.

## Abbreviations

ASHIP: Association of Statutory Health Insurance Physicians
DEGAM: German College of General Practice and Family Physicians
ECG: Electrocardiograms
GIM: Physicians in general internal medicine
GP: General practitioner
